# Systemic varicella-zoster virus infection in two critically ill patients in an intensive care unit

**DOI:** 10.1186/1743-422X-10-225

**Published:** 2013-07-08

**Authors:** Hideharu Hagiya, Maya Kimura, Toru Miyamoto, Fumio Otsuka

**Affiliations:** 1 Department of General Medicine, Okayama University Graduate School of Medicine, Dentistry and Pharmaceutical Sciences, 2-5-1 Shikata-cho, Kitaku, Okayama 700-8558, Japan; 2 Division of Dermatology, Tsuyama Central Hospital, 1756 Kawasaki, Tsuyama, Okayama 708-0841, Japan

**Keywords:** Corticosteroid therapy, Critically ill patients, Intensive care unit, Opportunistic infection, Varicella-zoster virus

## Abstract

Varicella-zoster virus (VZV) usually causes localized zoster in adults. However, in immunocompromised patients, it can cause systemic infection accompanied by complications such as pneumonia, encephalitis, and hepatitis. Although most of critically ill patients in intensive care unit (ICU) are immunologically compromised, they are usually not considered to be at risk for systemic VZV infection.

We report two cases of systemic VZV infection occurring in critically ill patients in an ICU. One patient was a 69-year-old man with *Streptococcus pneumoniae*-induced purpurafulminans, and the other was a 75-year-old woman with severe acute pancreatitis. During the clinical course in the ICU, characteristic vesicles with umbilical fossa appeared diffusely and bilaterally on their face, trunk, and extremities. VZV-specific IgG levels were confirmed to be elevated compared to that of the pre-onset, and a diagnosis of recurrent VZV infection was made in both patients. The patients were treated at the same ICU but did not coincide with each other; therefore a cross-infection was unlikely. They were treated with intravenous acyclovir, but the latter patient eventually died of respiratory failure.

VZV infection can cause a number of serious complications, and can lead to death in some patients. Early detection and proper treatment are needed to prevent the infection from spreading out and save the patients. It might be necessary to consider antiviral prophylaxis against VZV infection for a part of critically ill patients in ICU, although the effectiveness of this approach is yet to be established.

## Background

Varicella-zoster virus (VZV) has the potential to cause life-threatening infection in adults, especially in those who are immunocompromised. In addition to specific humoral immunity, cellular-mediated immunity is involved in protection against VZV infection [1-3]. Pneumonia, encephalitis, hepatitis, and disseminated intravascular coagulopathy (DIC) are well-known complications of systemic VZV infection, and the overall mortality rate has been reported to be high in such cases (approximately 30% among renal transplant patients) [4]. Previous studies have reported that transplant recipients, human immunodeficiency virus (HIV)-infected patients, and patients undergoing treatment with tumor necrosis factor-α inhibitors are at an increased risk of developing severe VZV infection [5-7]. Critically ill patients in the intensive care units (ICU) tend to be immunologically weak [8-12] and they are considered to be at a high risk for various opportunistic infections. However, systemic VZV infection has not been considered as such an opportunistic infection in ICU patients and its clinical picture has yet to be well assessed to date. We present 2 cases of systemic VZV infection occurring in critically ill patients in the ICU.

### Case presentation

#### *Case 1*

A 69-year-old man presenting with chills, rigor, stomachache, and vomiting was admitted to a hospital in a state of shock. He had undergone bone marrow transplantation and chemotherapy for multiple myeloma 4 years ago and had been administered prednisolone (PSL; 5 mg/day at that time) since then. Physical examination revealed systemic purpuric rash and laboratory tests showed renal impairment, liver disorder, a highly inflammatory state, lactacidemia, and DIC. Computed tomography (CT) demonstrated bilateral hilar shadows and a relatively small spleen. Under the diagnosis of septic shock, DIC, and acute respiratory distress syndrome (ARDS), the patient was admitted to the ICU. His Acute Physiology and Chronic Health Evaluation (APACHE) II score was 26, and his Sequential Organ Failure Assessment (SOFA) score was 10. *Streptococcus pneumoniae* (serotype 22) was detected in blood cultures and a diagnosis of purpura fulminans caused by *S. pneumoniae* was made. Despite an intensive care and antibiotic therapy, the purpura and necrotic lesions spread over his face and extremities, and eventually quadruple amputation was needed and actually performed on day 7. Hemodialysis was unavoidable because of renal impairment, and multiple blood transfusions were given due to prolonged pancytopenia. Methylprednisolone 1 mg/kg/day was administered for the treatment of ARDS, and the corticosteroid therapy was gradually tapered to 5 mg/day of PSL, which was the dosage prescribed before admission.

**Figure 1:**
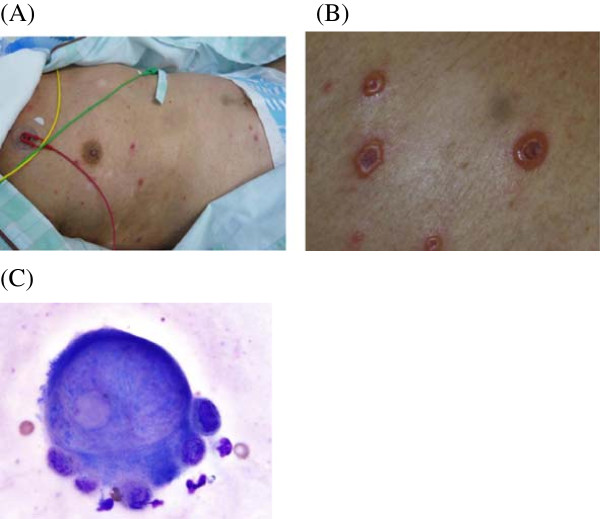
**Photos of characteristic vesicles seen in Case 1. (A)** The gross appearance of diffusely distributed vesicles. **(B)** The close appearance of vesicles; blistering with central umbilical fossa. **(C)** A giant cell obtained from vesicles (Giemsa stain, 1,000 power field).

On day 71, vesicles accompanied by umbilical fossa appeared diffusely and bilaterally on his face, trunk, and extremities (Figure 1A, B). Vesicles were in various stages, and a Tzanck preparation of the vesicles showed multinucleated giant cells (Figure 1C). Systemic varicella was suspected due to the appearance of these characteristic vesicles, and the patient was isolated and treated with intravenous acyclovir (reduced amount of 250 mg every 24 h due to renal insufficiency). There were no symptoms or findings suggesting pneumonia, encephalitis, or hepatitis. Although his serum VZV-specific IgM levels were not elevated, the IgG levels were found to be elevated from 13.2 to more than 128 for 3 weeks (Varicella-zoster IgG-EIA, Varicella-zoster IgM-EIA [SEIKEN], DENKA SEIKEN CO., LTD. Tokyo, Japan.). Acyclovir was administered for 7 days and the vesicles gradually formed clusters that were completed after 11 days. His history of VZV vaccination and VZV infection were unknown. Recent contact with VZV patients was unlikely because of his ongoing hospitalization in the ICU.

#### *Case 2*

A 75-year-old woman was admitted to our hospital because of abdominal pain. Her past medical history included coronary artery bypass grafting, aortic valve replacement, atrial fibrillation, diabetes mellitus, obesity (body mass index on admission was approximately 31), and psoriasis vulgaris. She had taken oral steroids for 4 years for the treatment of psoriasis vulgaris and was receiving 4 mg/day of PSL at the time of admission. Contrast-enhanced CT revealed an entirely swollen, poorly marginated pancreas, and she was diagnosed with severe acute pancreatitis. The patient was initially admitted to a general ward, however, respiratory failure worsened, and she was admitted to the ICU and intubated on the following day. Laboratory testing showed severe DIC, and APACHE II and SOFA scores at the time of ICU admission were 20 and 7, respectively.

A few days after the admission to the ICU, the patient’s consciousness was found to be worsened. Head CT revealed multiple cerebral infarctions. Otherwise, her subsequent condition was relatively stable. However, on ICU day 15, expiratory wheezing and vesicles appeared. By the next day, the vesicles distributed bilaterally on her back and buttocks that rapidly spread over her whole body. Systemic varicella was diagnosed due to the characteristic dermatological findings, and the patient was immediately started on intravenous acyclovir (500 mg every 8 h) and topical vidarabine. A liver disorder was detected 3 days after vesicles appeared; transaminase levels were elevated to maximums of 264 IU/L for aspartate aminotransferase and 157 IU/L for alanine aminotransferase. Although the patient's serum VZV-specific IgM levels were found to be not elevated either as the patient in Case 1, the IgG levels were elevated from 8.2 of one week before onset to 120.8 for two weeks. Under the diagnosis of VZV associated pneumonia and hepatitis, she was treated with 7 days of acyclovir. With treatment, respiratory wheezing and liver disorder disappeared, clustering was completed, and then patient left the ICU.

After that, she experienced a relapse of severe respiratory failure at the general ward. However, her family did not authorize intubation because of patient’s unconscious state at this time and eventually she died on hospital day 30. Although no autopsy was performed, chest radiography revealed deteriorated bilateral pulmonary infiltrations, and we clinically speculated that the patient died of VZV pneumonia or ARDS.

A detail of the two cases is summarized in Table 1.

**Table 1:** A summary of the two cases

	**Case 1**	**Case 2**
Sex	Male	Female
Age	69	75
Underlying disease	MM, bone marrow transplantation and chemotherapy	CABG, AVR, Af, DM, obesity, psoriasis vulgaris
Disease for ICU admission	*Streptococcus pneumonia*-induced Purpura fulminans	Severe acute pancreatitis
Complications after ICU admission	ARDS, Renal impairment, DIC, quadruple amputation, VAP	DIC, cerebral infarction
History of VZV vaccination	Unknown	Unknown
History of VZV infection	Unknown	Unknown
Recent contact with VZV	Not likely	Not apparent
Steroid use before admission	PSL, 5 mg/day	PSL, 4 mg/day
Steroid use after admission	mPSL, started with 1 mg/kg/day then tapered to PSL 5 mg/day	PSL, 4 mg/day
APACHE II score	26	20
SOFA score	10	7
Onset after ICU admission, days	71	16
Onset after intubation, days	71	16
Blood transfusion before onset	RCC, 78 units; FFP, 27 units; PC, 510 units	None
EIA-IgM (cutoff; 0.8)		
one week before onset	0.24	0.22
at the time of onset	0.15	0.15
after onset	0.52 (2 weeks)	0.47 (1 week)
EIA-IgG (cutoff; 2.0)		
one week before onset	13.2	8.2
at the time of onset	34.5	102.5
after onset	>128 (2 weeks after onset)	120.8 (1 week after onset)
Distribution of vesicles	Vesicles with umbilical fossa appeared diffusely and bilaterally on face, trunk, and extremities	
Time to cluster forming	11 days	7 days
Treatment, dose and duration	250 mg of acyclovir every 24 h intravenously for 7 days and 5 g/day of immunoglobulin for 3 days	500 mg of acyclovir every 8 h intravenously for 7 days and vidarabine topically
Outcome	Survived	Died 14 days after the onset

Abbreviations: *Af* Atrial fibrillation, *AKI* acute kidney injury, *APACHE II score* Acute Physiology and Chronic Health Evaluation II score, *ARDS* Acute respiratory distress syndrome, *AVR* Aortic valve replacement, *CABG* Coronary artery bypass grafting, *DIC* Disseminated Intravascular Coagulopathy, *DM* Diabetes mellitus, *EIA* Enzyme Immunoassay, *FFP* Fresh frozen plasma, *MM* Multiple myeloma, *mPSL* methylprednisolone, *PC* Platelet concentration, *PSL* Prednisolone, *RCC* Red blood cell concentration, *SOFA score* Sequential Organ Failure Assessment score, *VAP* Ventilator-associated pneumonia, *VZV* varicella-zoster virus.

## Discussion

Generally, critically ill patients have reduced cellular immunocompetence [8]. The overproduction of anti-inflammatory cytokines due to a highly inflammatory state causes immunosuppression, known as immunoparalysis [11,12]. Corticosteroid therapy, which suppresses cellular-mediated immunity, is frequently induced in various situations, and its long-term use often let the patients be immunocompetent state. Both patients presented here were in immunocompromised states due to their primary severe diseases and long period of corticosteroids administration. These conditions were considered being related to the onset of systemic VZV infection. Additionally, in Case 1, the relatively small spleen found by CT (spleen dysfunction is associated with reduction of humoral immunity [13]), the substantial blood transfusions, and the quadruple amputation were considered the other possible risk factors.

It was not clear whether these 2 cases were of disseminated zoster or varicella. The former is associated with multiple vesicular skin lesions in a generalized distribution affecting a number of distinct dermatomes that do not cross the midline, and the latter involves diffusely distributed several-staged vesicles. It is often difficult to differentiate the one to the other. Although a report of seemingly apparent varicella in an adult exists [14], atypical recurrent varicella without characteristic clustering have been described in immunocompromised patients [15,16]. We assumed that the presenting cases were varicella, rather than zoster, because of the dermatological appearance: diffuse and bilaterally distributed, but not closely aggregated.

As shown, systemic VZV infection can be fatal. Therefore, early detection and proper treatment is necessary. The vesicles or blisters are characteristic dermatological findings of VZV infection and can be the best indicator for diagnosis. However, VZV infection can progress without the appearance of typical vesicles, especially in immunocompromised patients [15,16]. In addition, specific antibodies are not always elevated in such patients [17,18]. Similarly, VZV pneumonia, the most frequent complication in adults, develops insidiously, usually after the onset of rash as seen in Case 2, and can progress to acute respiratory failure or ARDS. For these reasons, the early detection of systemic VZV infection is mandatory but often difficult. Accordingly, VZV prophylaxis may be required in some of immunosuppressed ICU patients to prevent fatal VZV infection. In bone marrow transplant recipients, the use of antiviral prophylaxis with low-dose oral acyclovir (600 mg daily) or ganciclovir (5 mg/kg 3-times weekly) is well established [17], but there is no guideline concerning prophylaxis for VZV infection in ICU. Acyclovir is also active against Human simplex virus (HSV) types 1 and 2, and acyclovir resistant HSV has been increasingly described as a problematic pathogen. Especially, the resistant organism has been isolated from a variety of immunocompromised patients (prevalence around 5%), including bone marrow recipients (prevalence reaching 30%), solid organ transplant recipients, and those with the acquired immunodeficiency syndrome (AIDS) [19,20]. VZV resistance to acyclovir has also been reported in patients with AIDS who had been given chronic acyclovir therapy [21]. Moreover, acyclovir has nephrotoxicity and drug interactions with the other frequently used drugs. We should cautiously determine the indicated patients if considering its use in the ICU setting.

Vaccination, another method of preventing VZV infection, is not possible for critically ill patients in ICU because it is the attenuated live vaccine and therefore contraindicated for immunosuppressed patients.

For VZV-infected immunocompromised patients, early high-dose acyclovir (e.g., 10 mg/kg 3-times daily) has been recommended [4]. In adult patients who progress to pneumonia or ARDS, corticosteroid therapy combined with acyclovir has been shown to reduce the duration of hospital and ICU stay [22,23]. Both patients described here were treated with 7 days of acyclovir. The therapy successfully treated the patient in Case 1; however, it was failed in Case 2. Although the relevance of the duration of acyclovir therapy to outcome is unclear, the patients should have been treated for longer period considering their impaired immunological state.

VZV is highly contagious; transmission occurs via direct contact with lesions or via respiratory droplets. Localized zoster is considered to transmit only by contact, while the virus can be infected by air-borne transmission in case of disseminated zoster. At present, there are no reliable guidelines available concerning the handling of VZV patients in the ICU. However, since many ICU patients are in a state of immunosuppression, we consider VZV patients should be isolated as soon as possible, and appropriate antiviral treatment should be initiated immediately to prevent the spread of the virus. As for the two patients, although they were treated at the same ICU, there was no chance of cross-contamination since their admission period was totally different and not overlapped.

## Conclusion

In summary, systemic VZV infection, although rare, can cause serious and fatal complications in critically ill patients in ICU. Early detection and proper treatment is critical for minimizing mortality and controlling infection. However, the typical dermatological findings of VZV infection and the elevation of specific antibodies can be absent in immunosuppressed patients. Prophylactic antiviral treatment, though not yet an established approach, should be considered, particularly in markedly immunosuppressed ICU patients.

## Consent

Written informed consent was obtained from the patient or family member for publication of this Case Report and any accompanying images. A copy of the written consent is available for review by the Editor-in-Chief of this journal.

## Competing interest

The authors declare that they have no competing interest.

## Authors’ contributions

HH mainly drafted the manuscript. MK, TM and FO helped to draft the manuscript. All authors read and approved the final manuscript.
